# Health Tract, No. 205

**Published:** 1864-08

**Authors:** 


### COOKING MEATS.

Every wife and mother owes it to herself, her husband, and her children, as well as to society at large, to prevent waste in every department of the household, whether provisions are cheap or dear, whether the husband is rich or poor; for waste is a crime against humanity, an insult to the bounteous Hand which "giveth us all things, riches to enjoy." On the other hand, a true economy is one of the wisest, and most ennobling of domestic virtues. A hundred careful experiments were made in England in reference to roasting and boiling meats, in order to ascertain the respective losses:

| | | | | | |
|---|---|---|---|---|---|
| Roasted chickens, lost | 15 | per ct. | Turkeys, lost........... | 20 | per ct. |
| Beef ribs and sirloins, | 19 | " | Mutton legs and shoulders, | 24 | " |
| Geese,.... ......... | 19 | " | Ducks, ................. | 27 | " |
| Boiled mutton legs,.. | 10 | " | | | |
| " beef, ........ | 15 | " | | | |
| " shoulder mutton, | 28 | " | | | |

Boiling beef saves more than four per cent over roasting. If a leg of mutton is boiled it loses ten per cent; if roasted, twenty-five per cent!

The fatter meat is, the greater the loss; it should be moderately fat, to make it tender; but there is an unprofitable fatness.

Eleven pounds of roast beef rib loses two pounds, and the bones one pound, so that of the eleven pounds bought, only seven pounds come to the table. Hence if roast rib-pieces cost in New-York, in April, 1864, twenty cents a pound at the butcher's stall, it is more than thirty-one cents a pound on the dinner-table.

It is philosophically true that one pound of clear roast beef is more concentrated than one pound of boiled beef, has less water in it, and hence may contain more nourishment; but the more concentrated food is the more unwholesome it is, not only because it requires a greater digestive power to convert it into pure blood, but the sense of sufficiency at meals is induced to a considerable extent by the bulk of what is taken, and if we eat concentrated food until there is bulk enough to remove the feeling of hunger, there is so much nutriment in it that nature can't extract it all in a perfect manner; hence there is not only too much nutriment for the wants of the system, but all of it is imperfectly prepared, and we really get less strength and less pure blood out of it, than if much less had been eaten, or it had been taken in a more bulky, or, if you please, in a more watery condition. This is the reason why dyspeptics and others eat a great deal, but they do not get strong. But if there is too much bulk, there is not enough nutriment, although a great deal is taken into the stomach. Porter and beer, for example, fill up the stomach, and seem to make persons fleshy, but there is but little nutriment and great bulk; but great beer-drinkers are never strong, are puffy.

